# Diagnostic value of triggering receptor expressed on myeloid cells-1 and C-reactive protein for patients with lung infiltrates: an observational study

**DOI:** 10.1186/1471-2334-10-286

**Published:** 2010-09-29

**Authors:** Ilias Porfyridis, Diamantis Plachouras, Vasiliki Karagianni, Anastasia Kotanidou, Spyridon A Papiris, Helen Giamarellou, Evangelos J Giamarellos-Bourboulis

**Affiliations:** 1 Department of Critical Care and Pulmonary Services, National and Kapodistrian University of Athens, 'Evangelismos' Hospital, Athens, Greece; 2 4th Department of Internal Medicine, National and Kapodistrian University of Athens, 'Attikon' Hospital, Athens, Greece; 3 2nd Pulmonary Department, National and Kapodistrian University of Athens, 'Attikon' Hospital, Athens, Greece

## Abstract

**Background:**

Differential diagnosis of patients with lung infiltrates remains a challenge. Triggering receptor expressed on myeloid cells (TREM)-1 is a neutrophil and monocyte receptor up-regulated during infection. The aim of this study was to evaluate the diagnostic accuracy of TREM-1 and of C-reactive protein (CRP) from patients with lung infiltrates to discern community acquired lung infections.

**Methods:**

68 patients admitted to a medical ward with acute respiratory illness were enrolled in the study. Neutrophil and monocyte TREM-1 expression were measured by flow cytometry, sTREM-1 by an enzyme immunoassay and C-reactive protein by nephelometry. Clinical pulmonary infection score was recorded.

**Results:**

34 patients were diagnosed with bacterial community acquired pneumonia (group A) and 34 with non-bacterial pulmonary disease (group B). Median serum TREM-1 concentration was 102.09 pg/ml in group A and lower than 15.10 pg/ml (p < 0.0001) in group B. Mean±SE neutrophil TREM-1 expression was 4.67 ± 0.53 MFI in group A and 2.64 ± 0.25 MFI (p = 0.001) in group B. Monocyte TREM-1 expression was 4.2 ± 0.42 MFI in group A and 2.64 ± 0.35 MFI (p = 0.007) in group B and mean±SE CRP was 18.03 ± 2 mg/ml in group A and 7.1 ± 1.54 mg/ml (p < 0.001) in group B. A cut-off of 19.53 pg/ml of sTREM-1 with sensitivity 82.6% and specificity 63% to discriminate between infectious and non-infectious pulmonary infiltrates was found. sTREM-1 at admission greater than 180 pg/ml was accompanied with unfavourable outcome.

**Conclusion:**

TREM-1 myeloid expression and sTREM-1 are reliable markers of bacterial infection among patients with pulmonary infiltrates; sTREM-1 is a predictor of final outcome.

## Background

Early diagnosis of lung infections remains a challenge. There is no gold standard for diagnosing microbial infection as clinical and laboratory signs are neither sensitive nor specific enough, and microbiological studies often remain negative. The presence of a new infiltrate on plain chest radiograph is considered indicative for diagnosing pneumonia, especially when is supported by clinical and laboratory findings. However it is difficult to differentiate a chest infiltrate of bacterial origin from a chest infiltrate of non-bacterial origin solely based on radiological criteria [1]. The diagnosis of infection is not always clear in the acute setting in patients with respiratory tract disease and a surrogate marker of infection would be a major benefit in the diagnostic armamentarium. Many inflammatory mediators and acute phase reactants, like C-reactive protein (CRP) and procalcitonin, have been described as reliable markers of infection; however none are specific enough, since they are also increased in non-infectious inflammatory conditions [2].

Triggering receptor expressed on myeloid cells (TREM)-1 is a recently described receptor on neutrophils and monocytes. It behaves like a pattern recognition receptor (PRR) since its activation leads to the release of pro-inflammatory cytokines, namely of tumour necrosis factor-alpha (TNFα) and of interleukin (IL)-8. Although its ligand is still unknown, activation is mediated by bacteria and fungi [3,4]. A soluble form of TREM-1, namely sTREM-1, is increased in the bronchoalveolar lavage (BAL) of patients with ventilator associated pneumonia (VAP) [5,6], and in the serum of patients with sepsis, with bacterial meningitis and with acute pancreatitis [7-12]. This same soluble form of TREM-1 seems to be increased in patients bearing non-infectious processes like peptic ulcer, inflammatory bowel disease, viral infections, malignant pleural effusions and chronic obstructive pulmonary disease (COPD) but also among patients after cardiac surgery or cardiac arrest. Increase of sTREM-1 seems particular prominent when the latter non-infectious states are complicated with systemic inflammatory response syndrome (SIRS) without infection [13-19].

Several published studies yielded contradictory results for the diagnostic and prognostic usefulness of TREM-1 and of sTREM-1 for infections [5,7,20-22]. The created impression is that more data are necessary to yield definitive results for its usefulness as a diagnostic and prognostic marker of community acquired pneumonia (CAP).

The aim of the present study was to define whether expression of TREM-1 on cell membranes of neutrophils (nTREM-1), of monocytes (mTREM-1) and serum sTREM-1 may help in the diagnosis of acute bacterial infections for patients admitted with a new pulmonary infiltrate or pleural effusion.

## Methods

### Study design

In this observation trial, all consecutive admissions to the Department of Critical Care and Pulmonary Services on predetermined and randomly selected emergency duty days were eligible. Inclusion criteria were: i) age above 16 yrs, ii) written informed consent; iii) acute respiratory illness and iii) presence of new pulmonary infiltrates or pleural effusion on chest x-ray or lung computed tomography. Exclusion criteria were: i) Human immunodeficiency virus (HIV) infection, ii) documented extrapulmonary infection, iii) neutropenia; and iv) oral intake of corticosteroids defined as any more than 1 mg/kg of prednisone for more than 1 month. The study protocol was approved by the Ethics Committee of the hospital and written informed consent was obtained from all patients within the first 12 hrs after admission.

Clinical, laboratory, and imaging data were recorded for each patient including: i) clinical presentation; ii) body temperature, iii) arterial blood gas, iv) peripheral blood cell counts, v) gram stains and cultures of all biological fluids obtained (blood, sputum, bronchial secretions, BAL, and pleural fluid); vi) imaging findings, vii) antigen serology (*Legionella *spp and *Streptococcus pneumonia *urinary antigen, serological testing for *Legionella pneumophila*, *Mycoplasma pneumoniae*, *Chlamydia pneumoniae*) and viii) in-hospital mortality. The severity of illness was assessed by calculating Acute Physiology and Chronic Health Evaluation (APACHE) II, Sequential Organ Failure Assessment (SOFA) and Clinical Pulmonary Infection (CPIS) scores at admission [23].

A diagnosis of community-acquired pneumonia (CAP) was established in any patient presenting with a combination of fever, cough and purulent sputum, shortness of breath, chest pain, and new consolidation on chest X-ray or computed tomography. The severity of pneumonia was assessed the first 24 hours of admission according to Confusion, Urea nitrogen, Respiratory rate, Blood pressure (CURB) index. Patients having two or more criteria were identified to have severe pneumonia [24]. Sepsis, severe sepsis and septic shock were defined according to current recommendations [25]. Pneumonia was considered to be absent when: i) an alternative cause for pulmonary infiltrate was established (e.g. pulmonary embolus) and ii) full recovery was achieved without antimicrobial therapy.

Pulmonary embolism was diagnosed according to current recommendations [26]. Lung cancer was ruled out based on histology and/or cytology specimens. Congestive heart failure was diagnosed according to American Heart Association [27], and interstitial lung disease according to American Thoracic Society guidelines [28]. All cases were evaluated by two clinicians blinded to TREM-1 and sTREM-1 results. Agreement about the diagnosis was achieved in all cases.

Patients with CAP were classified as having bacterial respiratory infection (*group A*). All other patients were classified as having non-bacterial respiratory disorders (*group B*). All patients assigned to group B were subject to chest computed tomography.

### Laboratory investigation

For the measurement of sTREM-1, mTREM-1, nTREM-1 and CRP 10 ml of peripheral venous blood were sampled after venipuncture of the antecubitul vein under sterile conditions on the day of admission and on days 3 and 7 of hospitalization. Seven ml were centrifuged and serum was stored in -80°C until assayed for sTREM-1. Three ml were collected into EDTA-coated tubes (Vacutainer, BD) for estimation of nTREM-1 and mTREM-1 expression. Briefly, red blood cells were lysed by ammonium chloride. White blood cells were labelled by phycoerythrin-conjugated anti-TREM-1 monoclonal antibodies (R&D InC, Minneapolis, USA) for 30 minutes in the dark. nTREM-1 and mTREM-1 expression were assessed after passage of labelled cells through a flow cytometer (Epics XL/MSL, Beckman-Coulter Co, Miami Florida) and expressed as the mean fluorescence intensity (MFI) with gating for neutrophils and for monocytes by their characteristic FS/SS scattering.

Determination of sTREM-1 was performed in duplicate by a developmental enzyme-linked immunoabsorbent assay according to the instructions of the manufacturer (R&D Inc, Minneapolis, USA). The lower detection limit and inter-day variation of the assay were 15.1 pg/ml and 5.23% respectively.

Measurement of serum CRP was performed by an immunoturbidimetric assay on Roche automated clinical chemistry analyzers and was expressed in mg/ml. CRP was used as a comparator due to its universal application in all studies of evaluation of biomarkers.

### Statistical analysis

Asumming that measured parameters between groups A and B differed by 50%, it was calculated that 30 to 40 patients should be assigned into each group to yield a difference at the 5% level with 80% power.

Values for nTREM-1, mTREM-1 and CRP are presented as mean ±SE; those of sTREM-1 are presented as medians and 95% confidence intervals (CI) or interquartile range (IQR). Comparisons between groups for nTREM-1, mTREM-1 expression and for CRP were done by ANOVA, followed by the Tukey's test for multiple comparisons. Comparisons of sTREM-1 between groups were done by Mann-Whitney U test after Bonferroni corrections for multiple comparisons. Comparisons of sTREM-1 between consecutive days within one group were done by Wilcoxon's signed rank test. Receiver Operator Curves (ROC) were designed to asses sensitivity, specificity, positive and negative predictive values for the estimated parameters to disclose infectious from non-infectious infiltrates. Patients were divided into two categories according to serum levels of sTREM-1 upon admission: those with sTREM-1 below or equal to 180 pg/ml; and those with serum sTREM-1 greater than 180 pg/ml. This concentration has been proposed as a threshold defining final prognosis in septic populations [22,29]. Since CAP is a common cause of sepsis, this threshold was considered of merit. Survival was assessed by Kaplan-Meier and comparisons were done by log-rank test. Correlations between severity scores and measured parameters were done according to Spearman. Probability values less than 0.05 were considered statistically significant. All statistics and graphs were done using the Statistical Package for the Social Sciences software version 17.0.0 (SPSS Inc, Chicago, IL).

## Results

**Figure 1:**
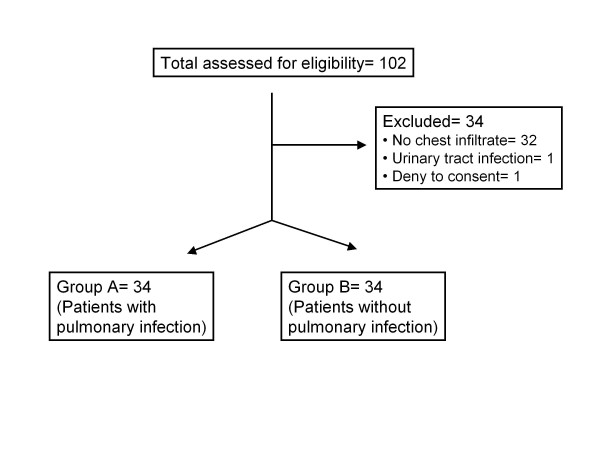
**Randomization chart of the study**.

The study flow-chart is shown in Figure 1. Demographic and clinical data of the patients are summarized in Table 1. Patients suffering from tuberculosis and enrolled in group B were presented with pleuritis.

**Table 1:** Demographic and clinical characteristics of 68 patients enrolled in the study.

	Group A (n = 34)	Group B (n = 34)	P
Age, (years, mean ± SD)	60.44 ± 3.58	65.83 ± 3.04	< 0.001
Male/female	19/15	21/13	ns
Diagnoses (n)	CAP (34)	• TB (2)	-
	• *Streptococcus pneumoniae *(4)	• Lung cancer (13)	
	• *Staphulococcus aureus *(3)	• Pulmonary embolism (6)	
	• *Haemophilus influenza*e (2)	• Congestive heart failure (5)	
	• *Pseudomonas aeruginosa *(2)	• Interstitial lung disease (6)	
	• Other (4)	• Other (2)	
pO_2_/FiO_2 _(mean ± SE)	240.18 ± 20.8	314.83 ± 15.9	0.006
SOFA (mean ± SE)	3.11 ± 0.43	1.66 ± 0.18	0.004
APACHE II (mean ± SE)	12.69 ± 1.06	9.4 ± 1.09	0.037
CPIS (mean ±SE)	5.38 ± 0.23	2.06 ± 0.25	< 0.001
Mortality [n (%)]	9 (26)	2 (5.8)	
White blood cells (mean ±SE)	14172.4 ± 1314.7	10470 ± 874.2	0.023

Group A (n = 34) consisted of patients with community acquired pneumonia (CAP) likely to be caused by extracellural bacteria. Seventeen had microbiological evidence of pulmonary infection, with isolation of the offending pathogens from sputum, blood or BAL samples (when bronchoscopy was performed). Seventeen patients were diagnosed with CAP on the basis of typical clinical and radiological presentation and good response to antibiotic therapy. Main radiological findings were right pulmonary infiltrate (10 patients); left pulmonary infiltrate (nine patients); and bilateral lung infiltrates (eight patients). Moreover, one patient presented with left pleural effusion; four patients had both right pulmonary infiltrate and right pleural effusion; and two patients had both left lung infiltrate and left pleural effusion.

Group B (n = 34) consisted of patients with non-bacterial respiratory disorders. Diagnoses were: lung cancer (13 patients); pulmonary embolism (six patients); interstitial lung disease (six patients); heart failure (n = 5); pulmonary tuberculosis (two patients); rheumatoid pleuritis (one patient); and Q-fever (one patient). Main radiological findings were: right pulmonary infiltrate (six patients); left pulmonary infiltrate (three patients); bilateral pulmonary infiltrates (11 patients); right pleural effusion (four patients); left pleural effusion (one patient); both right lung infiltrate and right pleural effusion (four patients); both left lung infiltrates and left pleural effusion (two patients); bilateral pulmonary infiltrates and left pleural effusion (one patient); and left pulmonary infiltrate and bilateral pleural effusions (one patient).

Among patients from group A with CAP nine (n = 9) died; six patients were admitted to the ICU and three were not admitted to the ICU due to relatives' denial. Mean age of patients not admitted to ICU was 80 years; the first two patients had a case-history of stroke and chronic heart failure; the third patient had a case-history of lung cancer. All three died from severe sepsis and multiorgan dysfunction syndrome (MODS). Mean age of patients admitted to ICU was 70 years; two patients had a case-history of aortic valve stenosis; two patients were under chronic intake of receiving corticosteroids; the fifth patient suffered from end-stage renal disease; and the sixth patient was suffering from hepatic failure due to alcohol intake. All six patients died from severe sepsis and multiorgan dysfunction syndrome (MODS). All patients in the ICU accomplished the clinical and radiological criteria for acute respiratory distress syndrome (ARDS) and were ventilated with the strategy of low tidal volume ventilation, according to current guidelines [30], with volume limited mode ventilation, low tidal volumes (about 6 ml/kg ideal body weight), a maximum of 25-30 breaths per minute, high positive end-expiratory pressure (PEEP 10 cmH_2_O) and a goal plateau airway pressure < 30 cmH_2_O. Among patients admitted in the ICU, two died on the second day post-admission; one died on the third day post-admission; one on the seventh day post-admission; one the eighth day post-admission; and one on the twentieth day post admission.

Concentrations of sTREM-1 and of CRP in sera of both groups and expression of nTREM-1 and mTREM-1 are given in Table 2. All four parameters were significantly greater in group A than group B.

**Table 2:** Concentrations of soluble triggering receptor expressed on myeloid cells-1 (sTREM-1) and of C-reactive protein (CRP) in the sera and expression of TREM-1 on neutrophils and on monocytes of 68 patients with pulmonary infiltrates.^§^

	Survivors	Non-Survivors	p	Group A	Group B	p
	sTREM-1 (pg/ml, median-IQR)					

Day1	28.13 (95.00)	189.61 (204.99)	0.028	102.09 (227.21)	< 15.10 (31.04)	< 0.0001
Day3	< 15.10 (39.45)	122.80 (231.05)	0.065	28.12 (263.74)	< 15.10 (21.10)	0.010
Day 7	< 15.10 (35.60)	52.30 (225.81)	0.055	12.96 (202.89)	< 15.10 (18.80)	0.022

	TREM-1 on neutrophils (MFI, mean ± SE)					

Day1	3.55 ± 0.53	4.18 ± 1.07	NS*	4.67 ± 0.53	2.64 ± 0.25	0.001
Day3	2.26 ± 0.25	3.40 ± 1.10	NS	3.14 ± 0.37	1.57 ± 0.20	NS
Day 7	1.46 ± 0.31	2.34 ± 0.95	NS	1.20 ± 0.37	1.60 ± 0.43	NS

	TREM-1 on monocytes (MFI, mean ± SE)					

Day1	3.24 ± 0.31	4.51 ± 0.91	NS	4.2 ± 0.42	2.64 ± 0.35	0.007
Day3	2.02 ± 0.33	3.42 ± 0.57	NS	1.50 ± 0.20	2.20 ± 0.43	0.001
Day 7	2.35 ± 0.25	4.25 ± 1.45	NS	1.76 ± 0.20	3.21 ± 0.43	0.003

	CRP (mg/dl, mean ± SE)					

Day1	11.6 ± 1.5	16.6 ± 3.8	NS	18.03 ± 2	7.1 ± 1.5	< 0.001
Day3	9.4 ± 1.4	12.3 ± 2.7	NS	12.2 ± 2.0	7.1 ± 1.5	0.05
Day 7	6.4 ± 1.1	9.7 ± 3.5	NS	7.2 ± 1.5	6.2 ± 1.6	NS

*NS: non significant

^§^Patients are divided into two groups; survivors and non-survivors; and into those with community-acquired pneumonia (group A) and into those with an infiltrate of non-infectious origin (group B).

**Figure 2:**
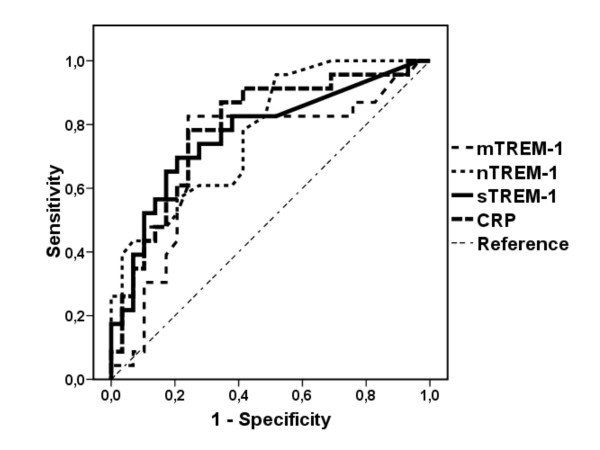
**Receiver operator curves (ROC) of serum sTREM-1, of TREM-1 expressed on neutrophils (nTREM-1), of TREM-1 expressed on monocytes (mTREM-1) and of serum CRP to discriminate between a pulmonary infiltrate of bacterial origin from a pulmonary infiltrate of non-bacterial origin**.

ROC of sTREM-1, nTREM-1, m-TREM-1 and CRP to differentiate whether a chest X-ray infiltrate is due to CAP or to a non-infectious process is shown in Figure 2. Area under curve (AUC) of sTREM-1 was 0.771 ± 0.068 (95%CI: 0.63 - 0.9, p = 0.001). Sensitivity and specificity to diagnose between a pulmonary infiltrate of infectious origin and a pulmonary infiltrate of non-infectious origin were 82.6% and 63% respectively at concentrations above 19.53 pg/ml. AUC of nTREM-1 and mTREM-1 were 0.778 ± 0.063 (95%CI: 0.65 - 0.9, p = 0.001) and 0.712 ± 0.07 (95%CI: 0.56 - 0.86, p = 0.009) respectively. Sensitivity and specificity to diagnose between a pulmonary infiltrate of infectious origin and a pulmonary infiltrate of non-infectious origin were 78.3% and 58.6% for nTREM-1 above 2.55 MFI. Sensitivity and specificity to diagnose between a pulmonary infiltrate of infectious origin and a pulmonary infiltrate of non-infectious origin were 82.6% and 75.9% respectively for mTREM-1 above 3.05 MFI. AUC of CRP was 0.789 ± 0.065(95%CI: 0.66 - 0.9, p < 0.001). Sensitivity and specificity to diagnose between a pulmonary infiltrate of infectious origin and a pulmonary infiltrate of non-infectious origin were 78% and 76% respectively at concentrations above 8.7 mg/ml.

Positive correlations were found between APACHE II scores and expression of TREM-1 on monocytes on day 1 (r_s_: +0.363, p: 0.010); and between APACHE II scores and sTREM-1 on day 1 (r_s_: +0.262, p: 0.043). No significant correlations were found between APACHE II scores and expression of TREM-1 on neutrophils on day 1 as well as between SOFA scores and any of the measured parameters on day 1.

**Figure 3:**
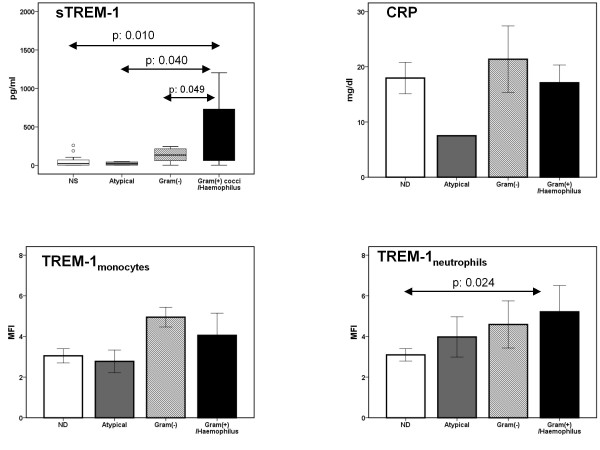
**Concentrations of sTREM-1 and of CRP in serum and expression of TREM-1 on monocytes and on neutrophils on the first day of acute respiratory illness of patients with new consolidation in chest X-ray**. Results are given as box plots for sTREM-1 and as means ± SE for the other parameters. ND: not defined. Statistically significant comparisons are indicated.

Correlations between serum levels of sTREM-1 and CRP and expression of TREM-1 on monocytes and neutrophils in relation to the identified causative pathogen of CAP are shown in Figure 3. Serum levels of sTREM-1 were greater among patients with CAP caused by Gram (+) cocci and *Haemophilus influenzae *than among patients with CAP caused by other pathogens.

**Figure 4:**
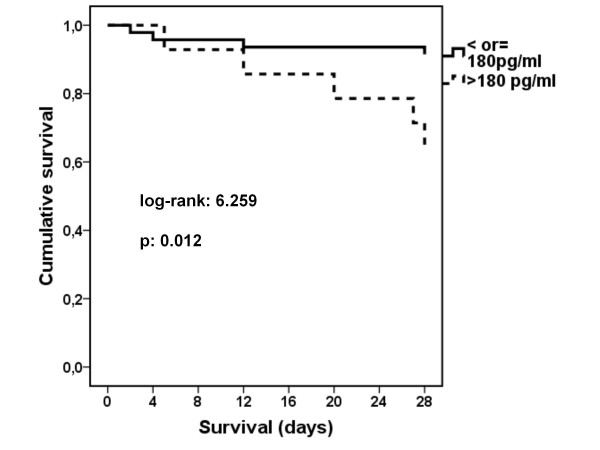
**Survival of patients with acute respiratory illness and with new consolidation in chest X-ray in relation to serum concentration of sTREM-1 on day 1**.

Death occurred in three out of 17 patients were no pathogen was defined (17.6%); in nil out of three patients infected by atypical pathogens (0%); in three out of seven patients (42.9%) infected by Gram-negative bacteria; and in three out of nine patients (33.3%) infected by Gram-positive cocci or *H. influenzae *(p: 0.034 between grouping according to pathogen). Survival of patients with sTREM-1 on day 1 below or equal to 180 pg/ml was prolonged compared with patients with sTREM-1 on day 1 above 180 pg/ml (Figure 4).

Concentrations of sTREM-1 and CRP in serum and expression of nTREM- and of mTREM-1 on days 3 and 7 are shown in Table 2. Estimated parameters were greater in group A than in group B. Median (IQR) sTREM-1 of survivors of group A was 90.52 (398.04) pg/ml on day 1; it was decreased to 17.20 (257.73) pg/ml on day 3 (p: 0.012 compared with day 1); and it was decreased to 10.40 (174.69) pg/ml on day 7 (p: 0.007 compared with day 1). Median (IQR) sTREM-1 of non-survivors of group A was 189.61 (186.73) pg/ml on day 1; it was unchanged on day 3 [144.07 (258.10) pg/ml; p: NS compared with day 1]; and it was unchanged on day 7 [59.03 (272.96) pg/ml, p: NS compared with day 1].

## Discussion

The results of the present study indicate that TREM-1 can be used as marker of bacterial infection in patients with lung infiltrates. sTREM-1, nTREM-1, mTREM-1 and CRP were comparable to their discriminating ability between a pulmonary infiltrate of infectious origin and a pulmonary infiltrate of non-infectious origin. sTREM-1 levels were decreased within the first 48 hours in patients with CAP with favourable outcome probably after the initiation of appropriate therapy followed by improvement of clinical symptoms. Finally, sTREM-1 levels above 180 pg/ml were an accurate independent predictor of in-hospital mortality from CAP.

Discrimination of the infectious or non-infectious origin of a pulmonary infiltrate remains an everyday clinical problem. CPIS was introduced for that purpose helping considerable in cases of ventilator-associated pneumonia (VAP) [23]. TREM-1 is a surface receptor on cells of the myeloid lineage. Activation of TREM-1 leads to the production of pro-inflammatory cytokines [9,31,32]. Binding of its ligand is possibly linked to the activation of several transcription complexes that synergize with NF-κB in order to elicit transcription of genes of pro-inflammatory cytokines [8]. sTREM-1 is the soluble counterpart of TREM-1 and it is probably shed in the systemic circulation from cell membranes of neutrophils and monocytes [7,33,34]. The physiologic role of sTREM-1 remains under question despite data support a probable anti-inflammatory role [31,35].

TREM-1 has been studied in patients with pneumonia, especially VAP [5,21,36-38]. Few data are available on the diagnostic role of TREM-1 and of sTREM-1 in patients with lung infiltrates. Our data are in agreement with observations from the study by Phua [36]. Their proposed sTREM-1 cut-off point was 163 ng/ml, which is different than the one we found. This may be result from the different method of assaying sTREM-1 the used being Western blotting.

The results of our study are in contrast to those of another study [37] that did not disclose any difference in nTREM-1 expression between patients with and without a bacterial lung infection probably due to the small number of patients included in that former study. El Sohl et al [38] reported elevated alveolar levels of sTREM-1 in pulmonary aspiration syndromes, but not in serum. However, serial plasma sTREM-1 levels were not obtained and the possibility that plasma levels might rise on subsequent days cannot be excluded.

Two recent studies [39,40] evaluated the diagnostic role of CPIS and of sTREM-1 in BAL fluid from patients with bilateral lung infiltrates in the intensive care unit (ICU). These studies reported controversial results. However authors did not measure sTREM-1 in serum on consecutive days.

The reported results of the present study are the first to our knowledge that evaluate the diagnostic value of TREM-1 among patients with lung infiltrates to discriminate CAP. They also disclose a relationship between levels of circulating sTREM-1 and causative pathogens. More precisely, infections caused by *Streptococcus pneumoniae*, *Sthaphylococcus aureus *and *Haemophilus influenzae *were accompanied by greater levels of sTREM-1 and by greater expression of TREM-1 on neutrophils than infections caused by other pathogens. Although it may be hypothesized that Gram-positive cocci and *H. influenzae *are strong inducers of TREM-1 expression, it should be emphasized that TREM-1 is one PRR, the exact agonist of which remains to be found [29,31].

A former study of our group [29] and another by Gibot et al [22] in heterogeneous populations of patients with severe sepsis of diverse aetiology investigated the role of early assessment of sTREM-1 as a determinant of final outcome. Results revealed that concentrations greater than 180 pg/ml are accompanied by survival benefit. The exactly opposing finding is reported here. This discrepancy may be explained by the enrolment of more homogeneous populations of patients, compared to these former studies [22,29], all suffering with CAP.

Our study presents two main limitations: a) no documented cases of CAP by *Legionella pneumophila*, *Mycoplasma pneumoniae*, protozoa or parasites were enrolled in group A; b) mortality in the CAP patient group was high probably due to the existence of severe co-morbid conditions.

## Conclusions

In conclusion, the presented results indicate that serum sTREM-1 and expression of TREM-1 on neutrophils and monocytes may serve as markers of CAP in patients with pulmonary infiltrates. Concentrations of sTREM-1 in serum are particularly increased in CAP caused by Gram-positive cocci and *Haemophilus *species. The real clinical value of sTREM-1 assay comes when TREM-1 levels are low, allowing the clinician to withhold empiric antibiotics until culture results are available, and thus eliminating unnecessary antibiotic exposure to the patient. And finally, early serum levels of sTREM-1 greater than 180 pg/ml in CAP are associated with unfavourable prognosis.

## Competing interests

Actual or potential conflict of interest: None. All the authors, Ilias Porfyridis MD, Diamantis Plachouras MD, Vasiliki Karagianni MD, Anastasia Kotanidou Ass. Prof. MD, Spyridon A Papiris Ass. Prof. MD, Helen Giamarellou Prof. MD and Evangelos J. Giamarellos-Bourboulis Ass. Prof. MD have no conflicts of interest to disclose related to this study. Evangelos J. Giamarellos-Bourboulis Prof. MD has received reimbursement for attending the 29^th ^International Symposium on Intensive Care and Emergency Medicine where participated as a speaker and unrestricted educational grants from ABBOTT Hellas SA; Wyeth Hellas SA; Sanofi-Aventis Hellas SA.

## Authors' contributions

IP participated in the study design, the enrolment of patients, the estimation of TREM-1, sTREM-1, CRP, the follow-up of patients and wrote the manuscript.

DP participated in the study design and in the estimation of TREM-1 and sTREM-1.

VK carried out the estimation of TREM-1.

AK and SAP participated in study design, and drafted the manuscript.

HG participated in study design and drafted the manuscript

EJGB coordinated the lab job, analyzed the data and drafted the manuscript.

All authors read and approved the final manuscript.

## Authors' information

IP is a MD in the Department of Critical Care and Pulmonary Services, National and Kapodistrian University of Athens, in 'Evangelismos' Hospital, Athens. DP is MD in the 4^th ^Department of Internal Medicine, National and Kapodistrian University of Athens, in 'Attikon' Hospital, Athens. VK is a MD of the 4^th ^Department of Internal Medicine, National and Kapodistrian University of Athens, in 'Attikon' Hospital, Athens. AK is an Assistant Professor MD in the Department of Critical Care and Pulmonary Services, National and Kapodistrian University of Athens, in 'Evangelismos' Hospital, Athens and expert in the management of critically ill patients. SAP is Assistant Professor MD in the 2^nd ^Pulmonary Department, National and Kapodistrian University of Athens, in 'Attikon' Hospital, Athens, Greece and expert in the field of respiratory diseases. HG is a Professor MD in the 4^th ^Department of Internal Medicine, National and Kapodistrian University of Athens, in 'Attikon' Hospital, Athens and expert in the field of infections and sepsis. And finally EJGB is an Assistant Professor MD in the 4^th ^Department of Internal Medicine, National and Kapodistrian University of Athens, in 'Attikon' Hospital, Athens and expert in respiratory infections and sepsis management.

## Pre-publication history

The pre-publication history for this paper can be accessed here:

http://www.biomedcentral.com/1471-2334/10/286/prepub
